# Pediatric kidney transplant and cardiometabolic risk: a cohort study

**DOI:** 10.1590/2175-8239-JBN-2021-0202

**Published:** 2022-02-28

**Authors:** Sara Mosca, Bárbara Gregório, Teresa Costa, Liane Correia-Costa, Conceição Mota

**Affiliations:** 1 Centro Hospitalar Universitário do Porto, Centro Materno-Infantil do Norte, Unidade de Nefrologia Pediátrica, Serviço de Pediatria, Porto, Portugal.; 2 Centro Hospitalar Universitário do Porto, Porto, Portugal.

**Keywords:** Allograft, Cardiometabolic Risk Factors, Dyslipidemias, Renal Insufficiency, Pediatric Obesity, Kidney Transplantation, Aloenxerto, Fatores de Risco Cardiometabólico, Dislipidemias, Insuficiência renal, Obesidade Pediátrica, Transplante de Rim

## Abstract

**Introduction:**

Patients with chronic kidney disease (CKD) are known to have increased cardiovascular risk but there are few data on the risk of pediatric kidney transplant recipients. We aimed to assess the impact of pre- and post-transplant overweight on allograft function and to characterize the evolution of several cardiovascular risk variables over time and their impact.

**Methods:**

A retrospective analysis of the records of 23 children/adolescents followed at a tertiary center after kidney transplant was conducted. Data on anthropometry and cardiometabolic variables were analyzed before transplant, six and 12 months after the transplant, and at the last follow-up visit. The impact of the variables on allograft function (glomerular filtration rate (GFR)) was estimated by creatinine-based revised Schwartz formula (Cr-eGFR) and was evaluated using nonparametric tests. Results: The 23 patients included in the study had a median age of 6.3 (4.4-10.1) years. Both systolic and diastolic BP z-score values significantly decreased between BMI groups [1.2 (-0.2 - 2.3) vs. 0.3 (-0.4 - 0.6), p=0.027 and 0.8 (-0.4 - 1.3) vs. 0.1 (-0.6 - 0.7), p=0.028, pre-transplant and at the final evaluation, respectively]. During follow-up, GFR values decreased (Cr-GFR: 68.9 (57.7-76.8) vs. 58.6 (48.9-72.9), p=0.033 at 6-months and at the end, respectively). Significant negative correlations between triglycerides and cystatin C-based eGFR (ρ=-0.47, p=0.028) and Cr-Cys-eGFR (ρ=-0.45, p=0.043) at the end of the study were found.

**Conclusion:**

Our study showed a high number of overweight children undergoing kidney transplant. A negative correlation between triglycerides and GFR was found, which highlights the importance of managing nutritional status and regular blood lipids evaluation after kidney transplant.

## Resumo

**Introdução:**

Sabe-se que pacientes com doença renal crônica (DRC) têm maior risco cardiovascular, mas há poucos dados sobre risco de receptores de transplante renal pediátrico. Visamos avaliar o impacto do sobrepeso pré/pós-transplante na função do aloenxerto e caracterizar a evolução de diversas variáveis de risco cardiovascular com o tempo e seus impactos.

**Métodos:**

Realizou-se análise retrospectiva dos registros de 23 crianças/adolescentes acompanhados em um centro terciário após transplante renal. Foram analisados dados sobre antropometria e variáveis cardiometabólicas antes do transplante, seis e 12 meses após transplante, e na última consulta de acompanhamento. O impacto das variáveis na função do aloenxerto (taxa de filtração glomerular (TFG)) foi estimado pela fórmula de Schwartz revisada e baseada na creatinina (TFGe-Cr), e avaliado usando testes não paramétricos. Resultados: Os 23 pacientes incluídos no estudo tinham idade média de 6,3 (4,4-10,1) anos. Valores do escore Z das pressões arteriaissistólica e diastólica diminuíram significativamente entre grupos de índice de massa corporal [1,2 (-0,2 - 2,3) vs. 0,3 (-0,4 - 0,6), p=0,027 e 0,8 (-0,4 - 1,3) vs. 0,1 (-0,6 - 0,7), p=0,028, pré-transplante e na avaliação final, respectivamente]. Durante acompanhamento, valores da TFG diminuíram [TFG-Cr: 68,9 (57,7-76,8) vs. 58,6 (48,9-72,9), p=0,033 aos 6 meses e ao final, respectivamente]. Encontramos correlações negativas significativas entre triglicerídeos, TFGe baseada na cistatina C (ρ=-0,47, p=0,028) e TFGe-Cr-Cis (ρ=-0,45, p=0,043) ao final do estudo.

**Conclusão:**

Nosso estudo mostrou alto número de crianças com sobrepeso submetidas a transplante renal. Verificou-se correlação negativa entre triglicerídeos e TFG, destacando a importância de controlar o estado nutricional e da avaliação regular dos lipídios sanguíneos após transplante renal.

## Introduction

Kidney transplantation has become the treatment of choice in both adults and children with kidney failure. In pediatric patients, early kidney transplantation was difficult but several advances have greatly improved outcomes in this age group^1^. Better quality of life, optimized growth, and longer patient and graft survival are among the advantages of kidney transplant compared with other renal replacement therapies^2^.

Patients with chronic kidney disease (CKD) are known to have increased risk of cardiovascular (CV) comorbidities and premature death throughout adulthood, thought to be due to the pediatric onset of end-stage renal disease (ESRD)^3^. The higher risk in CKD patients is believed to be related to several concurrent traditional risk factors, such as obesity, hypertension, dyslipidemia, and sedentary lifestyle. Additionally, other factors directly associated with the CKD status, namely hemodynamic or metabolic stress, inflammation, increased renin-angiotensin-aldosterone activity, and endothelial dysfunction, are all together probably enhanced by the immunosuppression regimens used in the particular setting of transplant recipients patients^4,5^. Thus, even after successful transplantation, there is an increased risk of CV disease. Besides the impact of donor-related factors and immunosuppression therapy, both pre- and post-transplant excessive weight gain, along with obesity-associated comorbidities, such as hypertension, dyslipidemia and insulin resistance, are increasingly recognized to significantly impact the renal graft function and morbidity of these patients. Moreover, obesity also seems to act as a risk factor for faster decline of allograft function, independently of the presence of other comorbidities^6,7^.

Many patients already present elevated blood pressure (BP) before the transplant, but an even larger proportion will develop hypertension afterwards, during follow-up^8-10^. Even in the absence of overweight, transplant patients are known to have an increased prevalence of both masked hypertension and nocturnal hypertension, with loss or reversed dipper pattern,, with an estimated one-third of patients being undiagnosed due to BP measurements performed in office only rather than ambulatory BP monitoring^5,11,12^. Additionally, a positive association between body mass index (BMI) and systolic blood pressure (SBP) has been reported to remain evident years after transplant^6,13^, with obesity contributing to even higher odds of masked and nocturnal hypertension after kidney transplant^5,14,15^.

In adults, dyslipidemia and insulin resistance are well-recognized risk factors for renal function decline, which are mainly associated with weight gain and immunosuppressants use after kidney transplant^16^. Studies in adults showed inverse relationships between glomerular filtration rate (GFR) and serum triglycerides and total cholesterol and also that, at 12-months post-transplant, total cholesterol was an independent risk factor for mortality^17-19^, but there are few data on the pediatric population after kidney transplant.

In the present study, we aimed to assess the impact of pre- and post-transplant overweight status on the allograft function in all kidney transplant pediatric patients followed-up at a tertiary center. We also aimed to characterize CV risk variables such as BP, blood lipids, and insulin resistance and their evolution over time following kidney transplant, and to evaluate their impact on allograft function.

## Material and Methods

### Study design and sample

In the present study, data of children and adolescents, aged 18 years or less at data collection (February 2020), who had undergone a kidney transplant and had their regular follow-up at the Pediatric Nephrology Unit, in Centro Materno-Infantil do Norte (CMIN), Centro Hospitalar Universitário do Porto (CHUPorto), were retrospectively evaluated. Two children were excluded for not having anthropometric data registered before kidney transplant. Finally, data of 23 children were analyzed; kidney transplants were performed between 2004 and 2019.

### Data collection and variables’ definition

A retrospective analysis of the clinical records of all included patients was performed. Data on sociodemographic features (sex, race, and age at kidney transplant and at last follow-up visit up to February 2020) were recorded. Data on clinical characteristics related to previous CKD (etiology, modality of renal replacement therapy) and kidney transplant (donor type, allograft survival time, immediate and late function of the allograft, therapeutic agents administered - immunosuppressant and other drugs) were also recorded.

Data on anthropometry (height, weight) and on cardiometabolic variables (office BP, fasting glucose, triglycerides, and total, low-density lipoprotein (LDL) and high-density lipoprotein (HDL) cholesterol) were recorded immediately before the transplant (pre-transplant), approximately 6 and 12 months after the transplant, and at the last follow-up visit. Height and weight were used for BMI calculation and BMI-for-age values were classified according to the World Health Organization growth reference data for BMI z-score into the following categories: non-overweight (-2SD to +1 SD) and overweight (> +1 SD, including also obese patients with > +2 SD)^20^. Fasting insulin was evaluated, and insulin resistance was determined at the last follow-up visit using only the homeostasis model assessment for insulin resistance (HOMA-IR).

Office BP determinations were assessed with validated automated oscillometric devices (Dinamap model Pro 300 series, Critikon^®^) with an adequately sized cuff on the right arm. When available, the average of the second and third measurements (with a 5-minute interval in between) at each occasion was used for analysis. Hypertension and elevated BP were defined according to the 2017 American Academy of Pediatrics guidelines.^21^ The presence of high BP was considered when patients presented either elevated BP or hypertension.

Overnight fasting venous blood analyses performed at baseline, approximately 6 and 12 months after kidney transplant and at the last follow-up visit (up to February 2020) were gathered. Data on hemoglobin, serum creatinine, urea, cystatin C (Cys), glucose, insulin, total cholesterol, HDL cholesterol, LDL cholesterol and triglycerides was analyzed, whenever available. Serum creatinine was analyzed using a calibrator for automated system (Roche Diagnostics) and serum CysC was measured by a particle-enhanced nephelometric assay (DADE - Behring, Siemens Company, European Format)^22^.

To estimate GFR in mL/min/1.73 m^2^, the following formulas were used^23^: creatinine-based formula - revised Schwartz formula (Cr-eGFR) = k x (height (cm)/serum creatinine (mg/dL), using a k constant of 0.413); cystatin C-based formula - Filler formula (Cys-EGFR) = Log (GFR) = 1.962 + [1.123 × log (1/cystatin C (mg/L))], and combined creatinine and cystatin C formula - Zappitelli combined formula (Cr-Cys-eGFR) = [(507.76 × e0.003×height(cm))/(cystatin C (mg/L)0.635 × serum creatinine (mg/dL)0.547) x 1.165].

### Ethics

The present study was approved by the Ethics Committee of Centro Hospitalar Universitário do Porto and complies with the Helsinki Declaration. Informed consent from children’s parents and verbal assent from children or adolescents for collecting information and biological samples was obtained.

### Statistical analysis

Statistical analysis was performed using IBM^®^ SPSS^®^ Statistics 26.0. Continuous variables are described as median and 25^th^ and 75^th^ percentiles and non-parametric testes were used, since data had a skewed distribution. Differences in independent continuous variables between groups were evaluated with Mann-Whitney test and paired variables were evaluated with Wilcoxon test. Differences in categorical variables were evaluated with Chi-square test. Bivariate associations between continuous variables were assessed by Spearman correlations. All p values were two-sided and considered statistically significant if <0.05.

## Results

The 23 children and adolescents included in the study had a median (25^th^-75^th^ percentile, P25-P75) age of 6.3 (4.4-10.1) years at kidney transplant and 17 (74%) were male. Baseline characteristics of pediatric kidney transplant recipients according to pre-transplant BMI status, non-overweight (n=15, 65%) and overweight (n=8, 35%), are shown in Table 1. Most of the patients (n=14, 60%) had a CAKUT as the cause of ESRD. Only one patient was submitted to a preemptive transplant; 59% (13) were on peritoneal dialysis, 14% (3) on hemodialysis, and 27% (6) on both therapies before kidney transplant for a median (P25-P75) time of 19 (7-51) months before kidney transplant. The participants had a median (P25-P75) Cr-eGFR of 68.9 (57.7-76.8) mL/min/1.73 m^2^ at 6 months post-transplant. All transplant recipients were initially maintained on standard immunosuppression therapy with tacrolimus (median dose 6.5 (5.49.6) mg/day), mycophenolate mofetil [median dose 600.0 (370.0-950.0) mg/day], and steroids [median dose 15.0 (10.0-15.0) mg/day]. At the end of the study, the immunosuppression therapy included for all patients a lower median dose of corticosteroids (2.5 mg/day) and tacrolimus 5.0 (4.4-6.0) mg/day.

**Table 1:** Baseline characteristics of pediatric kidney transplant recipients by pre-transplant body mass index classes

	All n=23	Non-overweight* n=15	Overweight* n=8	p
Male sex	17 (73.9%)	10 (66.7%)	7 (87.5%)	0.369
Age at transplantation (years)	6.3 (4.4-10.1)	7.8 (4.4-10.1)	6.1 (3.2-11.8)	0.846
**Primary kidney disease**				0.311
Non- CAKUT	9 (39.1%)	7 (46.7%)	2 (25.0%)	
CAKUT	14 (59.9%)	9 (53.3%)	6 (75.0%)	
Neurogenic bladder	2 (8.7%)	1 (6.7%)	1 (6.7%)	0.636
**Pre-transplant modality**				0.453
Preemptive	1 (4.5%)	1 (6.7%)	-	
Hemodialysis	3 (13.6%)	1 (6.7%)	2 (25%)	
Peritoneal dialysis	13 (59.1%)	10 (66.7%)	3 (37.5%)	
Hemodialysis / Peritoneal dialysis	6 (27.2%)	3 (20.0%)	3 (37.5%)	
Dialysis duration (months)	19.0 (7.0-51.0)	14.0 (5.8-51.2)	19.0 (10.5-51.0)	0.578
Deceased donor	19 (82.6%)	13 (86.7%)	6 (75.0%)	0.589
Follow-up after transplant (years)	7.4 (3.4-9.5)	8.2 (7.4-10-9)	3.2 (2.7-4.3)	<0.001
Anthropometric data**				
Weight (kg)	19.0 (14.4-27.0)	19.0 (14.4 - 26.4)	18.9 (13.9-37.4)	0.699
Height (cm)	108.0 (97.5 - 126.5)	117.0 (102.0 - 127.5)	103.5 (85.0 - 123.8)	0.258
BMI (kg/m2)	16.9 (15.6 - 19.6)	15.8 (15.0 - 16.9)	21.2 (17.8 - 23.2)	<0.001
z-score	0.4 (-0.3 - 1.3)	0.01 (-0.9 - 0.4)	1.4 (1.2 - 2.4)	<0.001
Office blood pressure data**				
SBP (mmHg)	105.0 (94.0 - 124.0)	105.0 (94.0 - 120.0)	110.5 (89.0 - 128.5)	0.698
z-score	1.2 (-0.2 - 2.3)	1.2 (-0.2 - 1.99)	1.4 (-0.2 - 2.3)	0.673
DBP (mmHg)	61.0 (51.0 - 74.0)	60.0 (52.0 - 77.0)	61.0 (41.8 - 69.8)	0.301
z-score	0.8 (-0.4 - 1.3)	1.1 (-0.3 - 1.8)	0.1 (-0.9 - 1.0)	0.114
High BP** *	9 (39.1%)	6 (40.0%)	3 (37.5%)	0.673

The values presented are median (25^th^ - 75^th^ percentile) or n (%).

* BMI z-score classes were defined according to the World Health Organization criteria; the overweight group includes overweight and obese patients^20^

** Anthropometric and blood pressure data collected at the last appointment before kidney transplant.

*** High blood pressure includes patients with elevated BP and with hypertension (stage 1 and stage 2), according to the 2017 American Academy of Pediatrics guidelines^21^ BMI: body mass index; BP: blood pressure; CAKUT: congenital anomalies of kidney and urinary tract; DBP: diastolic blood pressure; SBP: systolic blood pressure.

In the pre-transplant evaluation, the median (P25-P75) BMI and BMI z-score in the overweight group were 15.8 (15.0-16.9) and 1.4 (1.2-2.3), respectively. Patients in the overweight group had a shorter median follow-up time after transplant compared with the non-overweight patients [3.2 (2.7-4.3) vs. 8.2 (7.4-10.9), p<0.001]. Regarding office BP data, no differences on SBP, diastolic BP (DBP), and respective z-scores were found between the BMI groups. Nine (39%) patients presented high BP and were being treated with anti-hypertensive drugs before kidney transplant (Table 1).

**Figure 1:**
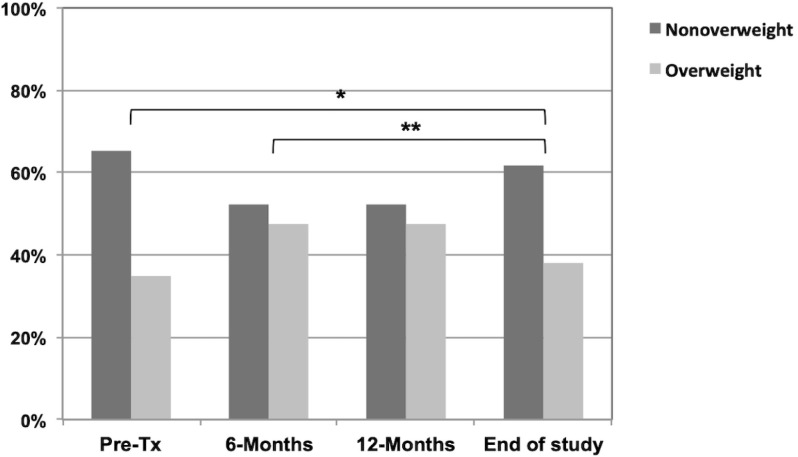
Distribution of patients by body mass index classes pre-transplant and at 6 months, 12 months and at the last follow-up visit after kidney transplant.

**Figure 2:**
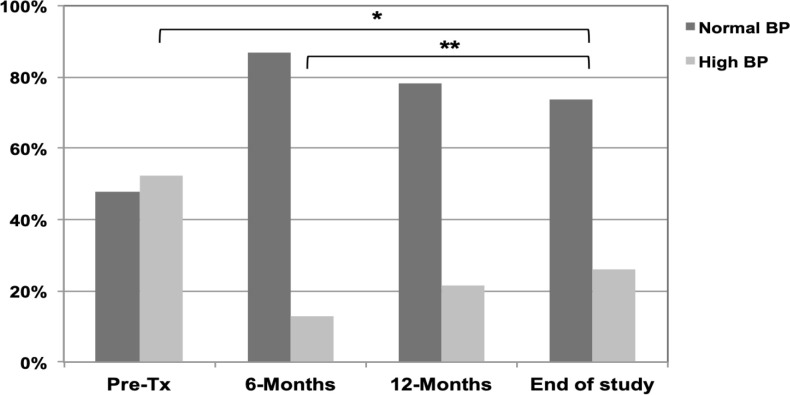
Distribution of patients by the presence of high blood pressure pre-transplant and at 6 months, 12 months and at the last follow-up visit after kidney transplant.

The distribution of patients by BMI classes during the study period is shown in Figure 1, by the presence of high BP is shown in Figure 2, and the variation of cardiometabolic parameters are reported in Table 2. The median BMI was higher at the end of the study compared with the 6-month evaluation [20.6 (18.0-24.4) vs. 18.0 (15.5-20.9), p=0.003] but the BMI z-score values was significantly lower [0.3 (-0.8-1.3) vs. 0.8 (-0.1-1.6), p=0.040], as was the prevalence of children classified as overweight (43.5 vs. 52.2%, p=0.019). A higher percentage of patients were overweight at the end of the study compared to the pre-transplant period, but the difference was not significant (35 vs. 43.5%, p=0.179); median BMI z-score values were similar [0.4 (-0.3-1.3) vs. 0.3 (-0.8-1.3), p=0.651]. The median SBP and DBP values were significantly higher at the end of the study compared to the 6-month-evaluation values, but the differences in SBP and DBP z-score values and the number of patients with high BP were not different. A lower percentage of patients presented high BP at the end of the study compared to the pre-transplant period, but the difference was not significant (52 vs. 13%, p=0.075) and SBP and DBP z-score values were significantly lower [1.2 (-0.2-2.3) vs. 0.3 (-0.4-0.6), p=0.027 and 0.8 (-0.4-1.3) vs. 0.1 (-0.6-0.7), p=0.028; for SBP and DBP, pre-transplant and at the end of the study, respectively]. Total cholesterol and triglycerides values were significantly lower at the end of the study compared to the 6-month evaluation after transplant [163.0 (139.8-187.5) vs. 149.0 (136.0-170.0), p=0.008 and 111.0 (77.3-139.5) vs. 86.0 (72.0-125.0), p=0.035, respectively]. No differences were found in the levels of LDL and HDL cholesterol or in the fasting glucose levels. Fasting insulin and HOMA-IR were only available for the last follow-up visit.

**Table 2:** Cardiometabolic parameters variation at 6 months, 12 months and at the last follow-up visit after kidney transplant

	Pre-Tx (n=23)	6 Months (n=23)	12 Months (n=23)	End of Study (n=23)	ΔPre-Tx-End of study	Δ6M- End of study
BMI (kg/m2)	16.9 (15.6 - 19.6)	18.0 (15.5-20.9)	16.6 (15.5-20.9)	20.6 (18.0-24.4)	0.004	0.003
z-score	0.4 (-0.3 - 1.3)	0.8 (-0.1-1.6)	0.5 (-0.5 - 1.6)	0.3 (-0.8-1.3)	0.651	0.040
Overweight*	8 (34.8%)	12 (52.2%)	11 (47.8%)	10 (43.5%)	0.179	0.019
SBP (mmHg)	105.0 (94.0 - 124.0)	102 (88-111)	103 (99-110)	110 (103-121)	0.404	0.001
z-score	1.2 (-0.2 - 2.3)	0.5 (-0.8 - 0.9)	0.4 (-0.2 - 1.04)	0.3 (-0.4 - 0.6)	0.027	0.765
DBP (mmHg)	61.0 (51.0 - 74.0)	57 (53-66)	62 (57-66)	63 (56-69)	0.578	0.020
z-score	0.8 (-0.4 - 1.3)	0.4 (-0.7 - 0.7)	0.4 (-0.4 - 0.6)	0.1 (-0.6-0.7)	0.028	0.627
High BP**	9 (39.1%)	3 (13.0%)	5 (21.7%)	3 (13.0%)	0.075	0.263
Total cholesterol (mg/dL)	NA	163.0 (139.8-187.5)	146.0 (128.0-169.0)	149.0 (136.0-170.0)	-	0.008
LDL cholesterol (mg/dL)	NA	91.0 (71.5-122)	83.5 (68.2-104.5)	83.0 (71.0-109.0)	-	0.705
HDL cholesterol (mg/dL)	NA	52.0 (37.8-62.5)	44.5 (36.4-53.8)	49.0 (38.0-57.0)	-	0.112
Triglycerides (mg/dL)	NA	111.0 (77.3-139.5)	100.5 (88.0-127.5)	86.0 (72.0-125.0)	-	0.035
Glucose (mg/dL)	NA	80.0 (75.5-85.5)	81.0 (70.2-87.8)	83.0 (76.0-87.0)	-	0.206
Insulin (µIU/mL)	NA	NA	NA	9.8 (6.2-14.3)	-	-
HOMA-IR	NA	NA	NA	1.9 (1.3-3.0)	-	-

The values presented are median (25^th^ - 75^th^ percentile) or n (%).

* BMI z-score classes were defined according to the World Health Organization criteria; the overweight group includes overweight and obese patients^20^

** High blood pressure includes patients with elevated BP and with hypertension (stage 1 and stage 2), according to the 2017 American Academy of Pediatrics guidelines^21^ BMI: body mass index; BP: blood pressure; DBP: diastolic blood pressure; HDL: high density lipoprotein; HOMA-IR: homeostasis model assessment of insulin resistance; LDL: low density lipoprotein; NA: not available; Pre-Tx: pre-transplant; SBP: systolic blood pressure.

**Figure 3:**
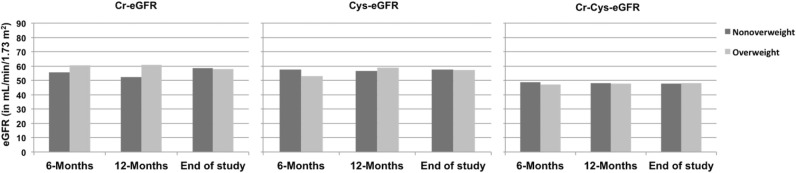
Median estimated glomerular filtration rate at the last follow-up visit according to body mass index classes at 6 months, 12 months and at the last follow-up visit after kidney transplant.

During the follow-up period, creatinine increased and Cr-eGFR values significantly decreased [68.9 (57.7-76.8) vs. 58.6 (48.9-72.9), p=0.033, at 6-months and at the end of the study, respectively]. Cr-Cys-eGFR also significantly decreased over the follow-up period [66.1 (57.1-71.9) vs. 47.6 (42.8-64.3), p=0.015; at 6-months and at the end of the study, respectively] (Table 3). The comparison of the actual median eGFR values (Cr-eGFR, Cys-eGFR and Cr-Cys-eGFR) evaluated at the last follow-up visit between BMI classes at 6 months, 12 months, and at the end of the study is shown in Figure 3. No differences were found between groups in neither of the GFR estimations considered.

**Table 3:** Renal function parameters variation at 6 months, 12 months and at the last follow-up visit after kidney transplant

	6 Months (n=23)	12 Months (n=23)	End of Study (n=23)	Δ6M-End of study
Creatinine (mg/dL)	0.7 (0.6-0.9)	0.7 (0.5-0.9)	1.1 (0.9-1.3)	<0.001
Urea (mg/dL)	50.0 (40.3-67.5)	46.0 (35.2-65.5)	63.5 (42.8-79)	0.060
Cystatin (mg/L)	1.2 (1.1-1.5)	1.4 (1.1-1.8)	1.5 (1.3-1.9)	0.136
Cr-eGFR (mL/min/1.73m2)*	68.9 (57.7-76.8)	71.3 (57.6-86.0)	58.6 (48.9-72.9)	0.033
Cys-eGFR(mL/min/1.73m2)**	73.6 (60.5-77.9)	62.6 (47.0-81.3)	58.3 (45.3-70.6)	0.071
Cr-Cys-eGFR (mL/min/1.73m2)** *	66.1 (57.1-71.9)	55.8 (48.8-77.7)	47.6 (42.8-64.3)	0.015

The values presented are median (25th-75th percentile).

* Cr-eGFR: creatinine-based estimated glomerular filtration rate (Schwartz formula)^23^

** Cys-GFR: cystatin C-based estimated glomerular filtration rate (Filler formula)^23^

*** Cr-Cys-based estimated glomerular filtration rate (Zappitelli combined formula)^23^

Spearman’s correlation between eGFR at the last follow-up visit and cardiometabolic variables during follow-up is shown in Table 4. A marginally significant correlation was found between SBP evaluated 6 months after transplant and Cys-eGFR (ρ=0.42, p=0.048) and between SBP evaluated 12 months after transplant and Cr-eGFR (ρ=0.46, p=0.046). Significant negative correlations between triglycerides at the end of the study and both Cys-eGFR (ρ=-0.47, p=0.028) and Cr-Cys-eGFR (ρ=-0.45, p=0.043) were found (Table 4). No other significant correlations were found between eGFR and the cardiometabolic variables evaluated.

**Table 4:** Spearman’s correlation between estimated glomerular filtration at the last follow-up visit after kidney transplant and cardiometabolic variables during follow-up

	Cr-eGFR (n=23)	Cys-eGFR (n=23)	Cr-Cys-eGFR (n=23)
**BMI (kg/m2)**			
6-Months	0.37	0.27	0.27
12-Months	0.23	0.18	0.12
End of study	0.02	-0.02	0.02
**BMI z-score**			
6-Months	0.31	0.07	0.12
12-Months	0.19,	0.11	0.06
End of study	0.07	-0.02	0.01
**SBP (mmHg)**			
6-months	0.43	0.42*	0.37
12-months	0.46*	0.43	0.39
End of study	0.16	0.39	0.30
**DBP (mmHg)**			
6-months	0.34	0.42	0.32
12-months	0.15	0.07	0.14
End of study	-0.07	0.21	0.16
**Total cholesterol (mg/dL)**			
6-months	0.01	-0.31	-0.15
12-months	-0.24	-0.17	-0.27
End of study	-0.04	-0.18	-0.10
**LDL cholesterol (mg/dL)**			
6-months	0.07	-0.08	0.001
12-months	-0.28	-0.16	-0.28
End of study	-0.19	- 0.19	-0.18
**HDL cholesterol (mg/dL)**			
6-months	0.06	-0.18	-0.16
12-months	0.10	-0.01	0.02
End of study	0.26	0.23	0.31
**Triglycerides (mg/dL)**			
6-months	-0.12	0.19	0.34
12-months	- 0.20	- 0.12	-0.11
End of study	- 0.34	- 0.47*	-0.45*
**Glucose (mg/dL)**			
6-months	-0.28	-0.23	-0.23
12-months	-0.23	-0.33	-0.33
End of study	0.09	0.06	0.02
**Insulin (µIU/mL)**			
6-months	NA	NA	NA
12-months	NA	NA	NA
End of study	0.01	-0.15	-0.05
**HOMA-IR**			
6-months	NA	NA	NA
12-months	NA	NA	NA
End of study	-0.02	-0.17	-0.06

* p value <0.05

BMI: body mass index; DBP: diastolic blood pressure; HDL: high density lipoprotein; HOMA-IR: homeostasis model assessment of insulin resistance; LDL: low density lipoprotein; NA: not available; SBP: systolic blood pressure.

## Discussion and Conclusion

In the present study, we described the impact of pre- and post-transplant overweight status on the allograft function of pediatric patients following kidney transplant. We found a very high percentage of overweight children/adolescents before the transplant (around 35%) and this percentage was about 43.5% at the end of the study. Although, a decline in eGFR values was observed over time, no significant differences were found in the median values of eGFR between the non-overweight and the overweight group at 6 months, 12 months, or end of the study. Concerning the other cardiometabolic variables analyzed, we found that BP z-score values were significantly lower at the end of the study compared to the pre-transplant evaluation. Triglycerides were significantly lower at the end of the study and negatively correlated with Cys-eGFR and Cr-Cys-eGFR at that point.

Overweight is a common problem after renal transplantation, which can be explained by several factors, including increased appetite and improved taste sensation associated with the resolution of uremia and the use of steroids, a reduction in dietary restrictions due to improved renal function, and a sedentary lifestyle and poor physical fitness^16^. A high percentage of children (35%) were overweight in before the transplant, and this number was even higher by the end of the follow-up period, although not significantly. The median BMI z-score remained stable during the follow-up period, with a slight increase in the 6-month evaluation. The prevalence of overweight/obesity found in our study was slightly higher than that reported in some previous pediatric studies^24,25^. This difference might be explained by the use of different reference values for BMI classification. In fact, the WHO definition of pediatric obesity usually yields the highest estimations of overweight and obesity compared to other definitions^26^. We should also consider that the prevalence of overweight and obesity in children is among the highest in Europe^27^, so it is to be expected that the nutritional status of the pediatric CKD population follows this trend, at least in part. Another 2013 study reporting the prevalence of overweight/obesity in the European pediatric renal replacement therapy population reported a prevalence of overweight/obesity of over 30% in transplant patients, especially in those over 6 years of age^28^, which is consistent with what we found in our study.

The finding of such a high prevalence of overweight among pediatric kidney transplant patients is particularly concerning since it has been increasingly acknowledged that overweight acts as an independent risk factor for decreased graft function and survival^6,24^. Investigating this association was our main goal because contradictory findings are reported in the literature, especially in children, for which strong evidence is scarce. Due to the size of our sample, the power may not have been enough to find a significant difference between non-overweight and overweight patients in the eGFR estimations considered, regardless of the formula used to estimate GFR (Cr-eGFR, Cys-eGFR and Cr-Cys-eGFR) and at different time points. Another possible explanation for this could be that, in our study, overweight patients had a shorter follow-up period than the non-overweight group. This might indicate that the problem of excessive weight gain in ESRD patients is more likely to affect those patients who have been transplanted in recent years. Previous studies with larger samples have found that GFR levels were significantly lower among overweight/obese patients^29^, while other authors have found no significant differences in terms the effects of overweight on renal function^6,29,30^, indicating the need for further studies in this age group.

CKD is known to be associated with increased CV risk in pediatric patients, which may be 3 to 5 times higher compared to their age-matched counterparts. Renal transplantation is thought to reverse some of the CV risk in these patients, but cardiometabolic factors continue to play an important role in the global risk of death in this population^31,32^.

The median SBP and DBP z-score values in our study were significantly lower at the end of the study compared to pre-transplant or even to the first evaluation after renal transplant, despite the difference at 6 months not being statistically significant. Hypertension is frequent among ESRD patients before transplant and usually persists after transplant, with a reported prevalence varying from 20% to almost 90%.^31,33,34^ In our study, high BP, including patients with high or normal-high BP, was present in more than half of the patients before transplant but in a significantly lower percentage of patients at the end of follow-up (around 13%). Our results regarding BP values seem to indicate that effective efforts have been made towards BP control after kidney transplant. This is especially relevant considering that a higher percentage of patients were overweight at the end of the study, which could have contributed to higher BP values.

The main reason for poor BP control after transplant seems to be the inappropriate use of anti-hypertensive drugs^35^. Multiple factors are known to be involved with post-transplant hypertension, and previous studies have reported a negative association between BP and GFR^12,13^. In our sample, we found a marginally significant positive correlation between SBP at 6 months and Cr-eGFR and between SBP at 12 moths and Cr-eGFR, which is in contrast with previous findings, and might be explained by the low number of children with high BP in our sample.^13^ We did not have data on ambulatory blood pressure monitoring that would have helped us better assess the association with GFR, since transplant patients are known to have an increased prevalence of both masked hypertension and nocturnal hypertension, with loss or reverse dipper pattern, which may have escaped us in the present analysis^5,11,12^. The benefits of using ambulatory blood pressure monitoring as a diagnostic tool to complement the study of hypertension and consequently increase graft survival have been shown in previous studies^11,12^.

The association between CKD and dyslipidemia is frequently reported in the literature^16,19^. After renal transplant, dyslipidemia is believed to contribute to a faster decline of the allograft function, especially when associated with proteinuria^36,37^. Evidence suggests that total cholesterol concentration 12 months after kidney transplant is an independent predictor of mortality^17^. Guidelines recommend early screening for dyslipidemia after transplantation, which should then be repeated at least annually^38,39^. A negative correlation between triglycerides and eGFR was found at the end of the study, which reinforces the importance of this recommendation. Similar findings have been reported in a previous study in pediatric kidney transplant patients, with no significant association between eGFR and both total and LDL cholesterol concentrations but a negative association with plasma levels of triglycerides^40^. Other previous studies failed to find an association between GFR and any lipid parameter after transplant, even when adjusting for age, BMI status, or primary kidney disease^40^.

Our study had several limitations. Although it was a prospective longitudinal study design after transplant, data were obtained from medical records and retrospectively analyzed. Besides, our study was a single center study and included a relatively small number of patients. We could not access data from patients transplanted in the pediatric age but who were older than 18 years and followed-up in adult facilities at the time of the study. Another important limitation was that we considered the end of the follow-up as a temporal mark for several comparisons, but in fact the follow-up time after kidney transplant varied among patients and a difference between BMI classes may have been present. Additionally, we did not have access to waist circumference measurements during follow-up, thus precluding the analysis of the impact of abdominal obesity in the associations studied. Another important limitation was the fact that BP evaluation was solely based on office BP, measured in an oscillometric automated device, with data on ambulatory BP monitoring only being available for about half of the patients, which was not reported in the present study. Ambulatory BP monitoring would have provided us with more complete information, allowing the diagnosis of masked and nocturnal hypertension and perhaps for a more reliable association with renal function.

Despite all limitations, our study showed that a high number of children and adolescents submitted to kidney transplant are overweight or obese. Considering the increasingly recognized impact of this risk factor on allograft function decline, our results should draw attention to the importance of better managing nutritional status in the high-risk group of ESRD patients. We observed a decline of eGFR over time, but we could not find differences in renal function between BMI groups probably due to a lack of power related to the sample size. Additionally, the negative association between triglycerides and GFR should also raise awareness to the importance of regular evaluation and management of blood lipids after kidney transplant. Although the number of studies on pediatric renal transplant have increased in recent years, more studies are needed to understand the best way to manage these patients. We believe that long-term studies, with larger samples and perhaps multicenter, are necessary to better assess the effect of obesity and its associated comorbidities on renal allograft function in children.

We hope to be able to continue to follow this sample in the future by adding ambulatory BP monitoring to their regular screening visits to monitor not only the evolution of kidney function but also the impact of cardiometabolic risk factors, which are expected to become more significant as time progresses.
